# Hand Hygiene Roles, Challenges, and Intervention Feedback from School Staff: A Qualitative Analysis, Belize, 2022–2023

**DOI:** 10.3390/ijerph22060835

**Published:** 2025-05-26

**Authors:** Anh N. Ly, Christina Craig, Dian Maheia, Yolanda Gongora, Vickie Romero, Rosalva Blanco, Allison Lino, Kelsey McDavid, Allison Stewart, Victoria Trinies, Alexandra Medley, Francis Morey, Russell Manzanero, Matthew Lozier, Kristy O. Murray

**Affiliations:** 1 Department of Pediatrics, National School of Tropical Medicine, Baylor College of Medicine and Texas Children’s Hospital, Houston, TX 77030, USA; 2 Division of Foodborne, Waterborne, and Environmental Diseases, Centers for Disease Control and Prevention, Atlanta, GA 30329, USA; 3 Belize Ministry of Education, Culture, Science, and Technology, Belmopan, Belize; 4 Division of Global Health Protection, Center for Global Health, Centers for Disease Control and Prevention—Central America Region, Guatemala City 01004, Guatemala; 5 Belize Ministry of Health and Wellness, Belmopan, Belize; 6 United States Public Health Service, Silver Springs, MD 20201, USA; 7 School of Medicine and Children’s Healthcare of Atlanta, Emory University, Atlanta, GA 30322, USA

**Keywords:** hand hygiene, behavior change, COVID-19, Belize, Central America, WASH in schools

## Abstract

Hand hygiene (HH) in school settings can reduce the spread of infectious diseases and student absenteeism due to illness. During the COVID-19 pandemic, the World Health Organization recommended HH as a public health measure to prevent disease transmission. Understanding school staff’s experiences with school-based programs is important for future program development and improvement. As part of a mixed-methods study, we conducted in-depth interviews in March 2022 with school administrators and teachers at 12 primary schools in Belize, selected based on high gaps in HH resources, to understand HH responsibilities, supplies, and challenges. An intervention was implemented to increase HH knowledge and practices among students, which included environmental nudges, supplemental provision of soap, and HH lesson implementation. Follow-up interviews were conducted in June 2023 among school administrators to garner feedback on the intervention. School staff described roles in teaching and managing HH supplies at both timepoints. The environmental nudges and HH lessons were perceived as helpful, but there were gaps remaining in HH practices, which may be partially influenced by practices and beliefs outside of school. Procurement of HH supplies remained a challenge at some schools due to financial constraints. The feedback from school staff will be valuable for the implementation of future hand hygiene programs in schools.

## 1. Introduction

Hand hygiene is critical to prevent the spread of respiratory and gastrointestinal diseases [1]. Globally, over 383,700 diarrheal-related deaths and over 355,500 respiratory-infection-related deaths were attributed to unsafe hygiene in 2019 [2]. Previous studies have shown that hand hygiene can reduce school absenteeism among students [3,4,5]. During the COVID-19 pandemic, the World Health Organization recommended hand hygiene as a public health measure to prevent the spread of infections [6]. Behavior change and adequate access to hand hygiene resources are necessary to foster and sustain hand hygiene habits. A systematic review showed that school-based programs can yield improvement in water, sanitation, and hygiene (WASH) knowledge, attitudes, and behaviors among students [7]. Since school is a setting for development and where children spend a large portion of their days, school staff can play a crucial role in educating and reinforcing hand hygiene practices.

In collaboration with the Belize Ministry of Education, Culture, Science and Technology (MoECST) and the Belize Ministry of Health and Wellness (MoHW), we initiated a mixed-methods study aiming to evaluate and improve hand hygiene practices among students at 12 primary schools in Belize using a multimodal behavior change intervention, including the implementation of environmental nudges and hand hygiene education. The quantitative data from this study showed gaps in hand hygiene resources and practices at the Belizean primary schools before and after the implementation of the behavior change intervention [8]. Despite the presence of hand hygiene supplies and environmental nudges, hand hygiene adherence remained low among students during the follow-up evaluation.

Previous studies in Bangladesh and the Philippines found improvement in hand hygiene following the implementation of environmental nudges in schools [9,10]. A related cluster-randomized trial in Bangladesh demonstrated that environmental nudges and hand hygiene education were equally effective at improving hand hygiene [11]. A review from 2021 highlighted systemic and behavioral factors such as cultural beliefs and government regulations having a significant influence on hand hygiene practices in Caribbean and Latin American countries [12]. A systematic review showed that environmental context, resources, knowledge, and goals are common determinants of hand hygiene practices in community settings [13]. This qualitative component of the study aims to understand the facilitators, barriers, management systems, and cultural and social environments in our pilot schools and their influence on student hand hygiene behaviors. Furthermore, we aim to understand the experience and challenges of the intervention from the perspective of school staff to inform future school-based hand hygiene programs.

## 2. Methods

### 2.1. Study Design

A survey on existing WASH services was distributed to all government and government-aided primary and secondary schools in Belize between December 2021 and January 2022. From the 221 schools that completed the survey, 12 schools (2 per district) with the largest self-reported gaps in WASH services were selected to participate in a pilot intervention and evaluation. In-person assessments were conducted at baseline in March 2022 and repeated at follow-up in June 2023 to measure the impact of the intervention. The intervention was implemented from October 2022 to May 2023. The baseline and follow-up assessments at the 12 pilot schools included in-depth interviews, facility assessments to document hand hygiene sources, observations of student hand hygiene practices, and hand dirtiness assessments. Additional information about the school selection, the timeline of the steps (survey, baseline, intervention, and follow-up) and the quantitative results were previously published; schools were classified as rural or urban by the Belize MoECST [8,14].

### 2.2. Intervention

The intervention included environmental nudges to encourage proper handwashing after using the restroom, distribution of soap for use in restrooms, and a workshop for hand hygiene lesson plan development for school administrators. The environmental nudges included footpaths leading from the toilet stalls to the nearest handwashing stations, handwashing reminders behind toilet stalls and above urinals if the school environment was not compatible with the footpaths, arrows pointing to the soap dispensers at the handwashing stations, and a message above the handwashing stations to remind students to use soap and wash hands for 20 s.

The lesson plan development workshop provided a collaborative environment for school administrators to creatively design hand hygiene lessons for all primary school grade levels, especially those with no specified learning outcomes related to hygiene. Following the workshop, the administrators were to distribute the lesson plans to teachers at their schools to adapt and implement in the classrooms. Due to the limited time between the workshop and the end of the school year, the lessons were to be implemented once. Additional details about the intervention were previously published elsewhere [8].

### 2.3. Interview Guide Development

The baseline interview guide was developed based on the researchers’ objectives of capturing key facilitators and barriers to hand hygiene in schools to inform the development of the intervention. Participants were asked about COVID-19 protocols and hand hygiene management systems, the availability of hand hygiene supplies, and the challenges associated with ensuring hand hygiene practices or adequate supplies. Due to time constraints, the questions were pilot tested at one of the schools and then revised for clarity prior to the remaining school visits.

The interview questions used at follow-up were designed based on the different intervention components and prior anecdotal experiences with the intervention from participating schools or field staff. The follow-up guide included questions about currently available hand hygiene resources, challenges with hand hygiene practices and supplies, feedback for intervention, and suggestions for future hand hygiene initiatives. These questions were pilot tested with Belizean enumerators and revised prior to the school visits. The interview guide is available upon request.

### 2.4. Data Collection

In-depth interviews (IDIs) were conducted by four enumerators (ANL, VR, RB, AL) from Baylor College of Medicine (BCM). All the enumerators received training in human-subject research and interview techniques (ANL and AL were U.S.-based epidemiologists with formal qualitative research training; VR and RB were Belizean nurses with prior experience in participant enrollment for research studies). At baseline, one administrator and one teacher were invited to participate in the IDIs at each pilot school to obtain different perspectives. Convenience sampling was used to select the school administrators based on the staff availability on the day of the visit. The administrators then assigned a teacher to participate in the interview. All the participants were selected and invited to participate in-person on the day of the visit. The baseline participants had no interaction with the enumerators prior to the interview.

During the follow-up visits, one administrator from each pilot school was invited in-person to participate in the interview; if unavailable, a teacher with knowledge of the school system was invited to participate instead. The sample size was reduced at follow-up as the research team determined that more data were collected than needed to reach thematic saturation at baseline. Administrators were selected instead of teachers because the administrators at most schools worked more closely with the research team during the intervention. Some interviewees participated at both baseline and follow-up; however, the data were not paired.

All the interviews were in English and conducted face-to-face on the school compound in a private area or inside a classroom (with no other school staff present). The interview duration ranged from 20 to 45 min. The enumerators explained to the interviewees the purpose of and procedures for the interviews and obtained verbal consent from all the interviewees prior to beginning the interviews. Non-participation was not documented. The interviews were audio-recorded and transcribed verbatim by a transcription service (TranscribeMe! at baseline and Transcription Panda at follow-up). Notes were not taken during the interviews to allow the enumerators to fully engage in the conversations. All the transcripts were deidentified prior to the analysis, stored on a secured BCM-approved drive, and only accessible to members of the study team. The transcripts were not returned to the study participants or any Belizean government officials. This study was approved by the ethics committees of the Belize MoHW and BCM (protocol H-49250).

### 2.5. Analysis

The deidentified interview transcripts were coded using MAXQDA 2020 (VERBI Software, 2020) [15]. The codebook was developed using both deductive and inductive methods [16]. Categories of codes were included based on the topics covered in the interview guide and additional common themes were added as they emerged from the interviews. The baseline and follow-up interviews were coded by the same primary analyst. The coded interviews were reviewed by one of the secondary analysts (4 at baseline and 1 at follow-up) to ensure the codes were applied consistently and all the relevant segments were coded. Discrepancies in code usage were discussed among all the coders early in the review process; additional code applications or modifications were made by the secondary analysts. The coded segments were analyzed using a thematic approach to identify and connect participant experiences across all the pilot schools. Illustrative quotes are embedded below.

## 3. Results

**Figure 1:**
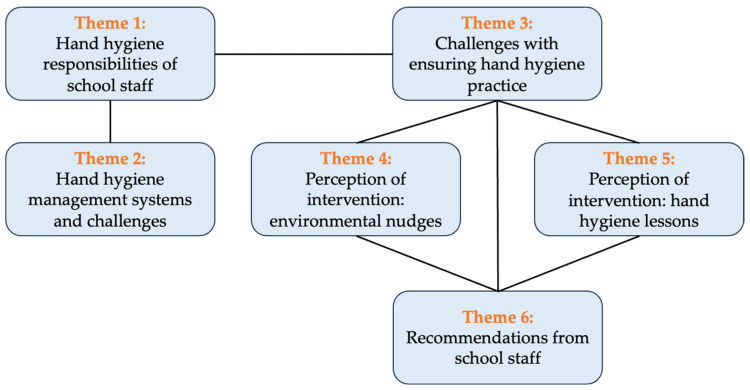
Themes that emerged from the staff interviews at baseline and follow-up.

During the baseline assessment, 22 interviews were conducted at the 12 pilot schools (10 rural and two urban), with 12 administrators and 10 teachers. The participant characteristics are summarized in Table 1. During the follow-up assessment, 10 administrators and one teacher participated in the interviews. One school was excluded from the follow-up interview because of unforeseen circumstances leading to the school not operating under normal conditions during the intervention and follow-up period. Of the 11 follow-up interviewees, 6 had participated in the hand hygiene lesson plan workshop. Many of the administrators at these pilot schools also had a teaching role during the school day (teaching administrators). The themes that emerged from the interviews are summarized in Figure 1 and in the following sections.

### 3.1. Theme 1: Hand Hygiene Responsibilities of School Staff

During the baseline interviews, school staff shared that they already had existing responsibilities related to hand hygiene even prior to the COVID-19 pandemic, such as restocking hand hygiene supplies, teaching students about hand hygiene, and reminding students to practice hand hygiene. At the start of the pandemic, schools implemented procedures to ensure a safe return to in-person learning for staff and students, which included more extensive hand hygiene practices, such as mandatory hand hygiene practice prior to entering the school compound. Staff shared that their roles since the pandemic had also expanded to monitoring and enforcing these new protocols during the school day and reinforcing hand hygiene concepts in the classroom.

At follow-up, staff were still responsible for some of these activities, such as teaching and reminding students to practice hand hygiene. Some schools still assigned times when staff would ensure all students washed their hands, primarily to help younger students develop good hand hygiene habits. One administrator shared that the emphasis on hand hygiene at school had been reduced as the COVID-19 pandemic became less of a concern in Belize.

*“… when the memorandum was sent to all primary schools and high schools and sixth form, immediately we start elaborating a plan of action in my school… [so that] the day that we reopened school again, there would be a way of how students will be safe in the washing of hands.”*

(Baseline, School 2, Teaching Administrator)

*“Well, ever since we came back to school, face to face, one of my responsibilities is to ensure that whenever children arrive at school, the first thing they do is wash their hands. So they can keep their hands clean before they enter the classroom.”*

(Baseline, School 4, Teacher)

### 3.2. Theme 2: Hand Hygiene Management Systems and Challenges

Hand hygiene infrastructure and management systems were similar at baseline and follow-up. At both timepoints, school staff shared that they received hand hygiene supplies such as soap and hand sanitizer from multiple sources, including the government, parents, and other donors. Each year, parents were asked to donate supplies for student and staff use at school. However, a common challenge was the communities’ lack of financial means to contribute to the supplies. Staff also mentioned purchasing supplies using the school fees collected from each student. Administrators and teachers sometimes purchased hand hygiene supplies with their personal funds if the school supplies ran out. Some students and staff brought their own supplies of hand sanitizer for their personal use.

All the pilot schools had access to a water source for handwashing. Some schools had access to water at no cost while others paid for their water usage. At follow-up, one school expressed concern about the cost of water. Some school staff also expressed challenges with the low water pressure or water shortages during the school year.

Administrators and staff had shared responsibility for managing hand hygiene supplies at school, including refilling or bringing soap bottles out from the classrooms when it was handwashing time for students; a few schools had wardens to support the management tasks. Some schools stored soap inside the classrooms instead of at the handwashing stations to prevent theft and misuse by students when left unattended. During the follow-up interviews, some administrators expressed that the donation of soap as part of the behavior change intervention helped to maintain supplies at schools, although the procurement of hand hygiene supplies overall was still a challenge. A few schools received both bar soap and liquid soap, and there were mixed preferences. Some staff preferred the bar soap because it lasts longer while other staff preferred liquid soap because it can be kept in the classroom.

*“…we get a few from the Ministry when it comes to liquid soap, Clorox, Flash. The parents are the ones [who] usually send donations or they send the child to school with alcohol, toilet paper, paper towels. We…send out letters to different organizations and they would supply us with some materials. Other than that, if we don’t have, then we have to use the fees collected by the school in order for us to purchase the supplies that we need.”*

(Baseline, School 10, Administrator)

*“The only problem the school is having right now in providing anything for the staff is finance. Honestly, that’s it because it’s not the availability of the product. It’s available. But we don’t have the finance to purchase it.”*

(Baseline, School 5, Teacher)

*“…we realized that…when…they [students] were not supervised they would waste…the liquid [soap]. And before the…week was over, we had to refill it…we met as a staff, and we agreed…that every teacher would… [have a] liquid container [in the classroom].”*

(Follow-up, School 2, Teaching Administrator)

### 3.3. Theme 3: Challenges with Ensuring Hand Hygiene Practice

At baseline and follow-up, school staff shared that students needed reminders to practice hand hygiene at school. Interviewees expressed that beliefs and behaviors reinforced at home can influence behaviors at school. One administrator mentioned during the follow-up interview that some parents no longer reinforced hand hygiene following the height of the pandemic. Another administrator shared that her students understood the importance of hand hygiene, but they tended to forget to practice it. At follow-up, some school staff shared that older students take more ownership of hand hygiene because they understand its importance and have established a routine. Other staff expressed that younger students follow hand hygiene guidance more deliberately.

*“Because we teach them here and they go home and practice something completely different. And then because of this COVID for two years [they’ve] been doing something far different from what we’re training them to do here…”*

(Baseline, School 7, Teaching Administrator)

*“…they’re [the students] aware of washing hands, and they know that they need to wash their hands… after they come from the bathroom… But sometimes I notice that… they actually… forget to wash their hands.”*

(Follow-up, School 4, Teaching Administrator)

### 3.4. Theme 4: Perceptions of Intervention: Environmental Nudges

School staff were generally supportive of the implementation of the hand hygiene nudges. A few staff expressed that the nudges were helpful in reminding students to wash their hands properly. Neither the schools nor this study sought feedback from students on their opinions of the nudges, but the interviewees believed that students were aware of the nudges. A few schools mentioned that the colorful footsteps and the pavers serving as stepping stones caught the attention of the younger students. Staff felt that some students understood the purpose of the nudges (i.e., placing soap below the arrow) but other students did not. For instance, one interviewee reported students treating the pavers as toys.

There were mixed responses on the impact of the nudges on student behaviors. Staff perceived some students were more attentive and aware of hand hygiene while others still needed reminders from the teachers to practice proper hand hygiene. One challenge of the painted footsteps shared during the interviews was the need to repaint for the footprint nudges to be visible. Some schools engaged students in the maintenance process to develop a sense of ownership.

*“They [the students] see it. They know it’s there. And they use it. I know for the pavers, they…like it. ‘Oh, little stones to hop on’… So, it’s just a means to get to where they need …they enjoy hopping from…stone to stone. It’s fun for them.”*

(Follow-up, School 3, Administrator)

*“… they [the students] have washed their hands, … some of them do take their time…there are a few that… I have to keep on reminding them … And some of them I noticed that [when] the soap is moved…I notice that they put it back where the arrow is.”*

(Follow-up, School 7, Teaching Administrator)

### 3.5. Theme 5: Perceptions of Intervention: Hand Hygiene Lessons

Staff shared that the workshop provided them with the opportunity to meet and collaborate with other administrators from different districts to develop interactive hand hygiene lesson plans. One staff member mentioned that it was challenging during the workshop to come up with lessons that would work for both rural and urban schools since the school settings varied.

Following the workshop, hand hygiene lessons were implemented in some classes in a variety of subjects, such as literacy or music. According to the interviewees, students were overall engaged with the lessons. The short timeframe available to implement the lessons following the workshop and before the school year ended was a common challenge. A few schools also had difficulty obtaining the necessary supplies to carry out the lessons due to time and resource constraints. In one small rural school with multi-grade classrooms, teachers found it challenging to adapt the lessons since they were designed for mono-grade classes. One administrator shared that some teachers prefer to plan their own lesson plans instead of executing lessons planned by others.

*“…it was great to listen to the different ideas from other colleagues across…the country. And it was good because it was not northern teachers only, but it was countrywide.”*

(Follow-up, School 6, Teaching Administrator)

*“So I came and I did it with the beat and the rhythm, and they love it, you know? ‘This is the way to wash your hands, wash your…’ and they started to sing all those things when they’re washing their hands. So I know…the message is there.”*

(Follow-up, School 8, Teaching Administrator)

### 3.6. Theme 6: Recommendations from School Staff

During the follow-up interviews, school staff suggested continuing with hand hygiene teaching and reinforcement inside the classrooms through interactive activities to foster hand hygiene knowledge and behaviors among students. A few staff suggested that future hand hygiene programs provide additional resources and continue with educational opportunities for staff, such as the lesson plan workshop. One interviewee mentioned that it would be beneficial to formally include hand hygiene in the curriculum for grade levels that do not currently have learning outcomes related to this topic.

*“And we want to focus more on the teaching in our lessons. Something that this year we were more concentrated on children practicing, but I believe that it has to be implemented… in every lesson, reminding children about [the] importance of having their hands clean.”*

(Follow-up, School 6, Teaching Administrator)

*“To provide schools with more supplies and to give and to educate…or have more workshops so teachers can get involved, and they can see that whatever is learned in the workshop, they can bring it out to the students.”*

(Follow-up, School 4, Teaching Administrator)

## 4. Discussion

This qualitative component of the study gathered perspectives from school staff to understand facilitators and barriers to student hand hygiene practices and school experiences with the behavior change intervention. We found that school staff have major roles in maintaining hand hygiene supplies and reinforcing hand hygiene practices. The interviewees were overall supportive of the behavior change interventions and reported student engagement with the nudges and hand hygiene lessons. During the interviews, school staff shared that hygiene-related social norms at home may play a significant role in hand hygiene practices at school. School administrators emphasized the importance of continuing with hand hygiene education at school.

As mentioned during the interviews, consistent access to hand hygiene supplies is a challenge at some schools due to financial constraints. Setting a designated portion of the budget for hand hygiene supplies may help ensure there is adequate funding to purchase the necessary supplies and encourage prioritization of hand hygiene among school staff. A study in western Kenya showed that equipping schools with a budget specifically for WASH supplies increased access to WASH resources at schools [17]. Coordination and partnership between key health stakeholders are critical to ensuring adequate and sustainable access to WASH resources and funding [18]. A framework for sustainable public health programs introduced by Schell et al. included funding stability, political support, partnership, and organization capacity as some key components [19]. In addition to access, the management of hand hygiene supplies in schools in Belize is an important factor that may influence practices. Some schools preferred to keep soap inside the classrooms instead of in restrooms to prevent theft or students wasting the soap. An evaluation of WASH practices in Belizean schools in 2011 showed that 72% of students washed their hands with soap after using the restroom when soap was kept at the sink, but none washed their hands with soap when it was kept in the classroom [20]. In addition to targeting behavior change to increase practices, future programs can consider promoting hand hygiene accountability among students, such as in a WASH intervention in Laotian primary schools where students were involved in the maintenance of the school environment [21].

While the findings from the in-depth interviews indicated that students at the pilot schools were engaged with the hand hygiene lessons, our survey of students showed no improvement in overall hand hygiene knowledge from baseline to follow-up [8]. This suggests that a one-time lesson was most likely inadequate to retain key hand hygiene knowledge among school-aged children. An educational program with a variety of teaching strategies and materials may be necessary to promote changes in student hygiene knowledge. For instance, a school-based intervention in Kenya on clean water and proper hygiene reported an increase in student knowledge after providing hygiene training and materials to teachers, forming safe water clubs, teaching students, and encouraging them to teach their parents [22]. A program focusing on soil-transmitted helminths in China reported an improvement in knowledge and handwashing among students after implementing a health education package, which included showing a cartoon, student discussions, pamphlets, and competitions [23]. It is important to also note that due to the short intervention timeframe, the hand hygiene lessons were not implemented consistently for all the grades at every pilot school, which may have influenced the perception of the interviewees at follow-up and the assessment of student knowledge at follow-up. A successful WASH educational intervention in Zambia involving repeated lessons during a 12-week period reported an increase in student knowledge and self-reported message transmission to their families [24]. A longer intervention period should be considered for school-based behavior change interventions.

Some school staff felt that there are still challenges associated with ensuring hand hygiene adherence among students, while others believed that their students practice hand hygiene properly following the intervention. Our observation of the hand hygiene practices at the schools showed no measurable improvement in hand hygiene adherence among students after the implementation of the intervention [8]. These findings suggest that staff perceptions of student behaviors may not always align with actual behaviors. One reason for this gap between perception and practice may be that staff do not always see students’ behaviors. Although there may be some monitoring of students, behaviors like proper hand hygiene practices after the use of the restroom may be difficult for school staff to monitor.

Although the environmental nudges were meant to serve as reminders for c hand hygiene, the in-depth interviews indicated that some students did not understand their purpose. Altering the design of the nudges based on student and school staff feedback is worth considering. Similar school-based interventions using environmental nudges in Bangladesh and the Philippines resulted in an improvement in student practices [9,10]; however, the local context of the schools may have played a role in the outcomes of our intervention in Belize. Note that these two studies were conducted in South and Southeast Asia, which may have different cultural norms and school functions compared to those in Belize. Additionally, we hypothesize that the reduced uptake of the intervention was related to a decrease in the public’s perception of the risk of COVID-19 and a decrease in hand hygiene emphasis compared to the earlier phases of the pandemic. The baseline, intervention, and follow-up evaluations took place during different stages of the pandemic. As time elapsed following the height of the pandemic, we observed a decrease in the emphasis on public health measures in the country, which may have influenced the interest in and awareness of hand hygiene. The implementation fidelity in our study was limited by the inconsistent installation and the constant maintenance needed to keep the nudges in good condition, which may have also influenced the impact of the intervention. The nudge-based study in the Philippines had the same challenge with the paint for their environmental nudges [10]. A more locally adapted pilot program with the participation of local members experienced with construction materials and school infrastructure should be considered to improve the impact and sustainability of the project. A few schools in the current project already involved students in the maintenance of the nudges, which was a creative method to promote leadership and ownership.

School staff shared that a common challenge to sustaining student hand hygiene practices at the pilot schools was the lack of hand hygiene awareness and practices in the community and the fact that students had not been to in-person school for nearly two years. Social and cultural influences can play a significant role in shaping health behaviors, especially among children, as they may model behaviors they observe from others in their surroundings, such as from parents or teachers. Similar to the school setting, hand hygiene practices in the community may be driven by knowledge, beliefs, and the accessibility of hand hygiene resources. A systematic review described social roles and influences as potential facilitators of and barriers to handwashing practices in community settings [13]. Another qualitative study showed that hand hygiene behavior change among primary school students is modified by a range of interpersonal and organizational factors, such as encouragement from adults, access to clean hand hygiene facilities, and facilitated opportunities for hand hygiene [25]. Future health interventions for students should consider social and structural determinants inside and outside the school environment. In the framework suggested by Appiah-Brempong et al., a hand hygiene educational intervention should consider individual-level factors such as knowledge, skills, and subjective norms, along with enabling social and physical environments, to promote behavior intention and adoption [26]. The inclusion of and partnership with the wider community may facilitate ideas and initiatives for ensuring adequate access to and use of hand hygiene supplies at schools.

This study had a number of limitations worth noting. Desirability bias may have been introduced into this study as the interviewees may have responded with what they believed to be favorable answers to the enumerators. This may be especially the case for the follow-up interviews as they were conducted by BCM personnel and schools were aware that the BCM team implemented all the interventions at their schools. Additionally, the interview responses may not capture the full experience of the intervention as school administrators may not be familiar with all the aspects of the intervention and how students engaged with them. For example, school administrators may not be aware of student hand hygiene practices after using the restroom to evaluate if the nudges had an impact on student hand hygiene behaviors. Furthermore, the interviewees’ perceptions of the intervention may not represent the views of all the staff at their school. Lastly, since only 12 schools were included in this study and they were selected based on self-reported gaps in WASH services in the national survey, the findings from this study cannot be generalized to the entire country of Belize or other schools outside of Belize.

## 5. Conclusions

School staff play a significant role in managing hand hygiene supplies and educating and ensuring hand hygiene practices among students. Although the multimodal intervention was widely accepted by school staff, there remained challenges in ensuring student hand hygiene practices. The interviews with school staff provided valuable feedback on the experience of the intervention and will be beneficial for designing future school-based programs to consider the local contexts. Future hand hygiene programs could benefit from including school staff, students, and community members in the design process to promote a culture of hand hygiene at school and outside of school. School-based programs should also be piloted at multiple phases for acceptability and feasibility to increase the fidelity of the programs.

## Figures and Tables

**Table 1:** Roles of the interviewees at the pilot schools at the baseline and follow-up evaluations.

School Number	Rural/Urban	Baseline	Follow-Up
School 1	Urban	Teaching administrator	Administrator *
		Teacher	
School 2	Urban	Teaching administrator	Teaching administrator
School 3	Rural	Administrator	Administrator *
		Teacher	
School 4	Rural	Teaching administrator	Teaching administrator
		Teacher	
School 5	Rural	Teacher	Administrator
School 6	Rural	Teaching administrator	Teaching administrator *
		Teaching administrator	
School 7	Rural	Teaching administrator	Teaching administrator *
		Teacher	
School 8	Rural	Teaching administrator	Teaching administrator
School 9	Rural	Administrator	Teaching administrator
		Teacher	
School 10	Rural	Administrator	No interview
		Teacher	
School 11	Rural	Teaching administrator	Teaching administrator *
		Teacher	
School 12	Rural	Administrator	Teacher
		Teacher	

* School staff participated in the hand hygiene lesson plan workshop.

## Data Availability

All relevant data are in the manuscript. Data sharing is not available to protect participant confidentiality.
